# What are the current barriers to effective cancer care coordination? A qualitative study

**DOI:** 10.1186/1472-6963-10-132

**Published:** 2010-05-20

**Authors:** Jennifer Walsh, James D Harrison, Jane M Young, Phyllis N Butow, Michael J Solomon, Lindy Masya

**Affiliations:** 1 Surgical Outcomes Research Centre (SOuRCe), School of Public Health, University of Sydney and Sydney South West Area Health Service, NSW Australia; 2 School of Psychology, University of Sydney, NSW Australia; 3 Discipline of Surgery, University of Sydney, NSW Australia

## Abstract

**Background:**

National cancer policies identify the improvement of care coordination as a priority to improve the delivery of health services for people with cancer. Identification of the current barriers to effective cancer care coordination is needed to drive service improvement.

**Methods:**

A qualitative study was undertaken in which semi-structured individual interviews and focus groups were conducted with those best placed to identify issues; patients who had been treated for a range of cancers and their carers as well as health professionals involved in providing cancer care. Data collection continued until saturation of concepts was reached. A grounded theory influenced approach was used to explore the participants' experiences and views of cancer care coordination.

**Results:**

Overall, 20 patients, four carers and 29 health professionals participated. Barriers to cancer care coordination related to six aspects of care namely, recognising health professional roles and responsibilities, implementing comprehensive multidisciplinary team meetings, transitioning of care: falling through the cracks, inadequate communication between specialist and primary care, inequitable access to health services and managing scarce resources.

**Conclusions:**

This study has identified a number of barriers to coordination of cancer care. Development and evaluation of interventions based on these findings is now required.

## Background

Cancer is a complex condition that often requires multiple interventions provided by a variety of health professionals over prolonged periods of time. In one UK study [1], it was found that patients, who had been treated for cancer for less than a one year period, saw 28 doctors on average, and this figure does not include other health professionals involved in their care. This adds complexity to health care service delivery and creates challenges for health professionals in both hospital and community settings to effectively work together to deliver high quality coordinated patient care.

Care coordination encompasses numerous aspects of health service provision including appropriate care that is timely and provided by a multidisciplinary team comprising of medical, nursing and allied health professionals. Other key elements intrinsic to care coordination include psychosocial assessment, suitable and timely referral, information provision and individualised treatment that considers each patient's needs and preferences [2]. A lack of coordinated care can lead to fragmented care, patients getting "lost" in the system and failing to access appropriate services, as well as more unplanned health utilisation [3,4].

There has long been emphasis in Australia [5] and overseas [6,7] on the need for strategic improvements in cancer care coordination, especially with increased pressure on cancer resources and services as a result of higher rates of cancer incidence, improved detection, treatment and survival. Despite a variety of initiatives introduced to improve care coordination, including the appointment of cancer care coordinators and strategies to enhance multidisciplinary care [8], there are still obstacles preventing its effective implementation.

Identification of barriers to care coordination is essential in order to implement strategies to address them on an organisational, team and individual level. Therefore, this qualitative study was undertaken to explore the views and experiences of patients, their carers and health professionals involved in cancer care regarding the key issues and challenges to achieving effective cancer care that is well coordinated.

## Methods

### Study population

Using purposive sampling [9], patients who were within 3 - 15 months of commencing treatment for cancer at a major metropolitan teaching hospital in Sydney, and their carers, were invited to participate in the study by their clinician. Advertisements were also placed in cancer support group newsletters and cancer clinics for participants from across New South Wales. The eligibility criteria included participants over 18 years of age that had been treated for any type of cancer, at any stage, and were cognitively able to participate. Health professionals involved in cancer care (surgeons, medical oncologists, cancer nurse coordinators and general practitioners) who the research team had previously collaborated with were approached directly, and participants then nominated colleagues who were also approached. In addition, a comprehensive list of New South Wales cancer care coordinators was obtained from the Cancer Institute New South Wales and those individuals were contacted directly by the research team. All participants were provided with a study information sheet and a consent form. Once consent was obtained, a member of the research team contacted the participant to organise attendance at a focus group or arrange a telephone interview.

The study was approved by the Human Research Ethics Committee of Sydney South West Area Health Service (RPAH Zone) and the University of Sydney.

### Data collection

This study aimed to explore participants' experiences of cancer care coordination in order to develop a richer and deeper understanding of the issues participants considered important and identify barriers to its successful implementation. In-depth, semi-structured interviews were conducted individually and in small focus groups with 20 patients, four carers and 29 health professionals and facilitated by two of the authors. Focus groups were undertaken in order to provide participants the opportunity to engage with others and share their care experiences, whilst individual interviews were offered to those who did not feel comfortable in group forums or would be unable to attend due to work commitments, illness-related problems or difficulties related to distance or transport. There were no apparent differences in themes arising in these different contexts.

Demographic data were collected using a study-specific questionnaire. Open-ended questions were developed on the basis of the literature by the research team (with multidisciplinary representation including a surgeon, psychologist, nurse and epidemiologist), as an interview guide to elicit the participant's experiences of care. For patients and carers, questions comprised the following themes (1) a description of the experience of their care; (2) what the term "care coordination" meant to them; (3) the management (coordination) of their care by health professionals; (4) what, if any, possible changes to the healthcare system to aid care coordination. For health care professionals, the themes were; (1) a description of what "care coordination" services involve; (2) an outline of the role they (health professionals) play in a patient's "care coordination"; (3) Any perceived deficiencies in "care coordination" processes; (4) the most important aspects of "care coordination"; and (5) a description of any differences in "care coordination" needs/processes between rural and regional areas. These broad themes were explored with each participant. Further probes were used as necessary to elicit greater detail dependent on each participant's response. Ongoing analysis was undertaken and recruitment continued until data had reached saturation [10] and no new themes or information on themes were emerging after three consecutive focus groups or interviews. Interviews and focus groups were audio-taped with the permission of participants.

### Analysis

Interviews and focus groups were transcribed and analysed with the aid of QSR NVivo 7 [11]. Prior to analysis, transcripts for each participant were de-identified to ensure confidentiality and to limit analytical bias among researchers [10]. A grounded theory [12] influenced approach was used to explore participants' experiences of cancer care coordination. The interview transcripts were reviewed line-by-line by three independent researchers who searched for initial concepts and themes. Themes were identified inductively, that is, rather than searching data for themes or threads from predetermined theory, the themes were developed from the data. Following this initial analysis, the process of axial coding was undertaken, whereby comparisons were drawn amongst the emerging themes. All classifications and comparisons were discussed until agreement was reached.

## Results

Twenty patients, four carers and 29 health professionals from across New South Wales were recruited into the study. It was not possible to determine the participant response rate as clinicians did not record the numbers of participants invited. All oncologists, surgeons, nurse practitioners and GPs who were approached consented to participate. Thirty-three percent of care coordinators approached consented to participate. Patient characteristics are shown in Table 1 'Patient and carer characteristics' and the health professional roles are illustrated in Table 2 'Health professional roles'.

**Table 1:** Patient and carer characteristics

		*n*	%*
**Gender^**	Male	10	42
	Female	14	58

**Age^**	35-44	1	4
	45-54	4	17
	55-64	14	58
	65+	5	21

**Type of Cancer**†	Bowel	14	70
	Breast	2	10
	Ovarian	1	5
	Uterine	1	5
	Leukaemia	1	5
	Lung	1	5

**Treatment**†	Surgery	5	25
	Chemotherapy	1	5
	Radiation therapy	0	0
	Multiple	14	70

*Where data is rounded percentages may not equal 100.

^Characteristics relevant to n = 24 patients and carers

†Characteristics only relevant to n = 20 patients

**Table 2:** Health professional roles

		*n*	%*
**Role**	Surgeon	3	10
	Medical Oncologist	1	3
	Family Physician	1	3
	Cancer Nurse Coordinator	2	7
	Nurse Practitioner	1	3
	Cancer Care Coordinator	21	72

* Where data is rounded percentages may not equal 100.

Qualitative analysis revealed the following barriers to effective care coordination.

### Recognising health professional roles and responsibilities

Several participants acknowledged confusion about the roles and responsibilities of the different members of the health care team involved in their care.

*"Particularly groups of patients who go from surgeon to medical oncologist, to radiation oncologist, they're often uncertain who's actually looking after them." *[Cancer Nurse Coordinator]

When patients and carers were asked about the responsibilities of individual members of their health care team, several recognised that there were so many people involved that they could not remember exactly who did what. Others conveyed some confusion when asked about individual responsibilities.

Researcher - "Who was in charge of informing you of the steps involved in your care?"

Carer - [Care coordinator X] was responsible for explaining things...."

Patient -"I would have said [Surgeon X]..."

Confusion surrounding the care coordinator role was especially apparent, not only with patients, but other health professionals. It was highlighted that such confusion had lead to limited referrals to care coordinators from the patients' health care team, as well as patients not utilising that resource when a referral was made.

*"The breast care nurse gave me her phone number in the pack of information, but she didn't say that I should call her if I had any questions or concerns." *[Patient]

Coordinator 1- "...not even using the word care coordinator - because I don't think that our patients actually know we are ----

Coordinator 2 - No, they think I'm the doctor's secretary....

Coordinator 3 - Yes, that's what they think too - they think I'm a secretary. A lot of them don't realize - if you say care coordinator, they may not understand that term....."

*"...the breast care nurse does a lot of the care coordination but she's called the breast care nurse, and the colorectal unit's got a care coordinator but she only looks after the surgical side of things. They may come and see the patient when they are having chemotherapy but that side of the journey is really coordinated, if you like, by the medical oncologist or the chemotherapy nurse treating the patient." *[Chemotherapy Nurse Practitioner]

*"That hasn't happened *(early referral to a care coordinator)*in my position for a number of reasons, I think it's partly because the position is not well understood, and the value of the position is not understood." *[Care coordinator]

Another issue that was raised about care coordinators was the potential for their role to cause conflict with other health care professionals, such as GPs, who often have a longstanding relationship and familiarity with their patients and see themselves as the coordinator of their patients' care.

*"....there's been a lot of resistance to coordinators because GPs see themselves as the primary care coordinator - 'Why do you need someone else to do the job?' What I've found though, is once the coordinator helps them with a patient and they actually discover, 'Well, this is what the benefit is to my patient and to me', generally they're on the phone a lot more. So if they've got something that's difficult they'll make that referral and it will happen." *[Care coordinator]

Care coordinators noted that they wanted to promote their role within the community to create a greater understanding and increase patient referrals, but often lacked time to do this properly and also feared an opposite problem, being overwhelmed with referrals.

*"Again, the difficulty with my particular position is that I would be out much more proactively advocating and advertising the role to GPs and communities but the position is time-limited and that raises an expectation which would not be possible to meet." *[Care coordinator]

### Implementing comprehensive multidisciplinary team meetings

Health professionals noted that the multi-disciplinary team (MDT) meeting is now considered an integral component for providing coordinated, collaborative care. The MDT meetings aim to allow different clinicians to discuss treatment options, and offer alternatives to treatment paths that have been chosen. They should also assist in clarifying roles and simplify the clear communication of roles and responsibilities to the patient. These meetings however, are not being implemented as consistently or successfully as recommended. There were several reasons presented as to why this is so. These included time constraints, lack of support for meetings, logistical issues in trying to get all members of the MDT together at the one time, large geographical distances between team members, staff shortages in key disciplines such as oncology, a lack of administrative support for these meetings and dominant personalities limiting open discussion.

*"Some of our specialists feel that everyone should be discussed, but time constraints mean that they can't be, so we need to be selective. We need to stick to those patients when the discussion is determining whether or not they are to have further treatment." *[Care coordinator]

*"....we have four surgeons in our hospital, one of them comes to most meetings; one of them comes to a fair few; one comes to hardly any and one has never been, but at least we know he is never going to come." *[Care coordinator]

*"Our MDT meetings sometimes have dominant personalities that just keep talking - dominating the conversation. Sometimes they're sharing ideas, but you have to make sure that it is the presentation of the case and allowing equal contribution, not somebody talking about a case and riding rough shot over everybody else!" *[Surgeon]

### Transitioning of care: Falling through the cracks

Patients who received treatment at different centres reported a lack of communication and effective referral between these centres, particularly from large urban centres of expertise back to the local services from which patients sought support. Health professionals stated that patients often don't get referred back into local support services they need when they have sought treatment elsewhere and it is then left to the patient to present themselves to local hospitals or support services.

*"Communication gets messy, no one quite knows what's going on and a patient can fall through the gaps in terms of the treatment plan. Or they're not able to come here, and then things get messy again as people near them geographically haven't got to know them. That's a particular issue with things like mesothelioma, where people come here because there are experts here, but then it's vital to try and communicate back to their local centre and visa versa." *[Care coordinator]

*"If people access specialist care outside of our area then that's obviously a difficult part in either picking them up when they come back, or follow-on care and again for a rural area there's a number of metro-based specialists that hold clinics in X, so the path is people get referred to them cause they can see them locally but then they're taken away to access care..." *[Care coordinator]

Limited links between private health care and community sectors was another barrier discussed. Many clinicians stated that there was a lack of communication and coordination between the public and private sectors.

### Inadequate communication between specialist and primary care

The barrier most frequently mentioned by health professionals was inconsistent, delayed and incomplete communication amongst the health care team, particularly between family physicians and specialists which inhibited the delivery of coordinated patient care. Family physicians noted the delay in delivery of diagnostic findings, treatment, complications and follow-up hindered their ability to provide appropriate advice to patients when they approached them in-between visits to their hospital healthcare team, or when they had completed surgery or other adjuvant treatments. This situation was often exacerbated by not knowing who to contact in the hospital care team to get the information required. Family physicians also identified that they lost touch with their patients while they were having specialist treatment.

*"...The general practitioner *[GP] *is probably one of the most important, but often the last to be notified of different things. So if the patient is discharged from here on Monday and has a problem on Tuesday, rocks up at the GP's office, they have no idea what's gone on... We're aware of that and we try to minimise those events, but there's always problems with communication." *[Care Coordinator]

*"I think probably one of the real challenges for GPs is that we often lose contact with our cancer patients during the treatment phase. They're so busy being seen by the oncologist and the hospital team, the radiotherapist, the surgeon and so on, we tend to lose touch with them. And that can be for quite a long period of time. It's not uncommon and then they come back to you and its awkward then trying to pick up the pieces." *[General Practitioner]

*"...some patients, especially if they're older, they often don't know a lot about their own care plan, and if there hasn't been a good record of that in their notes, if there hasn't been good communication from the hospital, it can be really difficult to try and get your head around that. I mean you have to go back through various letters and try to work out 'When was this patient diagnosed? What stage are they up to in their illness? When were they last seen?"' *[General practitioner]

### Inequitable access to health services

#### A) Rural/regional disadvantage

There was consensus amongst all participants regarding the inequity of health service access across Australia. Concerns were expressed regarding the acute lack of available support to rural patients, due to limited health care providers and local community support services in these areas. Lack of available specialist health care was seen as a major factor in delayed diagnosis.

*"I was living with a friend 275 kilometres from the only known (to me) treatment centre - site X. Results indicated some form of cancer but were inconclusive. I then had two trips to site X, 550 km return journey from home for bone marrow tests."* [Patient]

*"People with cancer & their carers in rural communities are forced to face additional issues such as; relocation to metropolitan centres for lengthy specialist treatment, being away from family & friends & other support networks whilst undergoing treatment and meeting hefty travel and accommodation costs."* [Patient]

*"I had a patient recently, who ended up needing a biopsy, and the GP couldn't do it, the referring specialist in the town didn't do it, so then the patient had to come back to us *[metropolitan centre]." [Care coordinator]

*"There is a lot of help at site X but none out here in the bush." *[Patient]

*"So it's delayed care, delayed diagnosis, delayed treatment, and they get worse prognosis because of that, I'm sure they do." *[Surgeon]

*"The remoteness and the lack of resources is a problem throughout the entire country, and with the increasing skills shortage, I'm not sure how that's going to be remedied. Trying to provide the same service to Mrs Jones here *[in Sydney] *as Mrs Brown out at Broken Hill may not be possible with the limited resources and skilled personnel..." *[Surgeon]

Location also influenced the roles and responsibilities of health professionals. In metropolitan and regional areas, most care coordinators were tumour specific and able to provide quite specialised care. In contrast, care coordinators in rural areas often had to deal with multiple tumour streams including palliative care, spreading their resources over a greater number of specialist areas and patients.

*"I think that's the hard part for rural people, it's not streamlined, it's not just breast *[cancer], *it's breast, colorectal or whoever turned up in your centre, you're doing everything." *[Cancer Nurse Coordinator]

*"...one girl, X, said sometimes she takes on all the disease groups, because she's the only one that covers quite a large area, and she said some people would benefit from the service, but they die before she sees them." *[Care coordinator]

#### B) Public and private care differences

Whilst both patients and clinicians recognised that patients could enter the private hospital system without delay, there was perceived to be a much lower level of supportive and psychosocial care available in the private, compared to the public system.

*"....to get in and get treatment, to have surgery, you can get into the private system pretty quickly, the waiting lists in the public system are quite long. But then there's a turnaround where once you've had your surgery, the level of *(private) *support after that doesn't exist. So there are no dieticians or physios or allied health support to help patient's post-surgery, whereas in the public hospital system there are." *[Cancer Nurse Coordinator]

*"Oh, you are a public patient? And you're getting all that attention? That's good..." *[Patient]

*"Yes, in the private I didn't get any [psychosocial support]..." *[Patient].

### Managing scarce resources

Participants identified an under-supply of various health care professionals throughout Australia, but what was of particular concern was the situation in terms of general practitioners and surgeons. The current general practitioner (GP) shortage within Australia was raised as a barrier to effective care coordination that could adversely affect patients' care across their entire cancer journey. This shortage places pressure on existing GPs who have to manage large patient volumes. It was highlighted that although the shortage is Australia-wide, it was critical in rural areas.

*"But, normally if you want to see a GP, you book in a month in advance. I can't get in to see a GP when I'm sick, it just can't happen....." *[Patient]

*"We are forever saying that they need to get into their GP and they say "I can't get into them", so I ring the GP or the receptionist, and get them in on the day or they end up in accident emergency. So, I think we need more GPs up here." *[Care Coordinator]

The increasing number of patients accessing cancer services limited the care and support health professionals felt they could provide their patients. Limited time and resources also tended to shift the care of patients to alternate health professionals. The GP shortage in particular adds to the workload of care coordinators, who are often contacted by patients with health concerns which should be addressed by their GP. Care coordinators are actually perfectly placed to assist and alleviate issues regarding GP and surgeon access, especially as a point of contact and an information source. However, these positions are also limited in supply and therefore over-worked. Care coordinators in rural and regional areas who were not specialised and therefore dealt with all cancer streams felt particularly pressured and time poor when attempting to identify and address their patients' needs.

*".....having the time for those people just to sit down and talk, which as busy professionals, it's just getting harder and harder to devote the appropriate amount of time to individual patients that we would like and that they deserve. So things become very like - problem, fix it, move on." *[Surgeon]

*"...if there's no care coordinator or similar type person, those issues are either not resolved and that's bad or the patients seek alternate help themselves from their GP or other people." *[Surgeon]

*"Care coordinator X and I both have a number of different tumour groups that we are supposed to look after but I spend all my time looking after two...., I sort of pick up the worst ones but really, that's as much as I can do..." *[Care coordinator]

## Discussion

**Figure 1:**
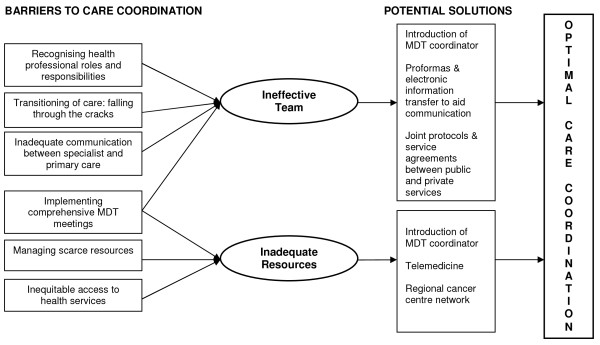
**Barriers Framework**.

There is an abundance of literature on the need for, and benefit of, care coordination for people affected by cancer [3,7,8]. However there is a paucity of research identifying current barriers to successful care coordination as identified by those ideally placed to recognise them; patients, carers and health professionals. The identification of current obstacles has the potential to guide the development of future initiatives to improve quality coordinated health care. Data from this study suggest that these barriers can be classified into two main categories. Firstly, those barriers that are a result of the performance of an ineffective team which includes; recognition of health professional roles and responsibilities, falling though the cracks: transitioning of care and inadequate communication between primary and secondary care. Secondly, those barriers that are the result of inadequate health care resources, including managing scarce resources and inequitable access to health services. The 'implementing comprehensive multidisciplinary team meetings' barrier extends across both of these categories as inadequate MDT meetings are the result of both the performance of an ineffective team and inadequate health care resources. Barriers identified from this research and their categorisations are illustrated in Figure 1.

Multidisciplinary care, with regular multidisciplinary team meetings at its core, is considered the foundation for providing high quality, coordinated cancer care [13]. Ideally, meetings should involve clinical, primary, community and allied health professionals. Regular MDT meetings facilitate efficient treatment planning, simplify referral processes between professionals (including care coordinators) and avoid unnecessary or duplicated examinations and investigations [14]. This current study has highlighted that there are numerous limitations to the successful implementation of multidisciplinary team meetings (MDTs). Sub-optimal attendance of key personnel such as surgeons and oncologists, the extended length of the meetings and issues associated with rural locality have been documented as barriers to their successful implementation [15]. Care coordinators in this current study also highlighted difficulties in the administration of MDT meetings. This supports the results of a study by Kelly et al [16] of nurse specialists, which found that the additional burden of organising and managing the MDT meeting often restricted their ability to provide a comprehensive service to their patients within the constraints of a normal working week. The introduction of an MDT coordinator to organise and facilitate the meetings (as opposed to the care coordinator) is one suggestion to address these ongoing issues.

Undertaking regular meetings with the entire MDT would assist in addressing the pervasive lack of understanding and recognition of health care professional roles; especially that of the cancer care coordinator. The role of the cancer care coordinator is a unique and distinct one, encompassing both clinical and non-clinical aspects of care. Care coordinators support patients by guiding them along their treatment pathway, ensuring access to appropriate information and support services [17], as well as acting as a key contact. Considering that the care coordinator role was introduced to facilitate patient care coordination, and as it encompasses vital patient support functions, these findings suggest a need for greater understanding within the MDT of this professional role. Patients also need to be made fully aware of the functions of their care coordinator. If a care coordinator is not part of a patient's health care team, then it is essential that measures are in place to ensure that the patient, as well as their health care team, understands who is responsible for their coordination throughout the differing phases of care.

The introduction of an MDT coordinator to communicate patient discussions and decisions would also assist in timely and complete patient information transfer between specialists and general practitioners. Following diagnosis, specialist services take the lead during active treatment, yet GPs offer a long-term continuity of care that is rarely equalled in the hospital system. Many patients have long established relationships with their GP and therefore may consult them for advice in-between specialist appointments or upon completion of surgery or other adjuvant treatments. A Scottish study found that in the year following diagnosis of early breast cancer, women consulted their GPs more than ten times; double their consultation rate with specialists [18]. A lack of information transfer between GPs and specialists has been reported in a Dutch study involving breast cancer patients. Only one in five GPs had received a report from the specialist by the time their patient had initiated a consultation. Almost half of the GPs surveyed described the communication process as being too slow; with a quarter describing the information provided as insufficient [19]. This current study has highlighted a persistent need to develop and evaluate interventions to improve the provision of information between primary and specialist care. There is a movement towards electronic communication given that speed of information transfer is essential [20]. One possible strategy is the development of GP designed proformas; however the processes, content and formats used for information transfer need to be agreed upon by all stakeholders [21]. For example, Tattersall et al [22] investigated stakeholder views and designed a proforma letter to facilitate communication between cancer specialists and GPs, and this has the potential to be adapted for electronic transmission.

True multidisciplinary care provided in one health facility as one seamless service is rare [23]. Therefore, in order for care to be integrated, larger specialist centres must create links with smaller centres. Although visiting specialists and teleconferencing provide such a link in some centres, a more systematic approach is needed [5] to address this barrier. The absence of links between the private hospital setting and community sectors was also an important issue noted by participants in this current study. Private patients were identified as lacking access to information and referral to appropriate support services once they were discharged from hospital. In fact, Chisholm et al [24] also found that private patients were more likely to be unaware of available support services compared to public patients. Coordination amongst these different sectors, settings and teams needs to be organised more proactively. The introduction of clear referral pathways, guidelines, joint protocols or service agreements between private and public supportive care services may help address this issue [25,26].

The location of large specialist centres primarily in metropolitan areas present physical and economic challenges to patients residing in rural and remote areas including distance, limited transport and the cost of travel. Specialist cancer services are limited to major cities often forcing patients living outside these areas to travel long distances to receive treatment. A recent population health survey reported that 27.6% of adults in rural areas had difficulties accessing health care compared to 12.5% in urban areas [27]. Participants in this current study also highlighted limited access to healthcare as one of the main barriers to early cancer diagnosis. This is despite directives that emphasise that patients should be encouraged to present early; be diagnosed promptly and progress speedily through the system [6]. Indeed, studies in several countries have reported that more remote patients were less likely to be diagnosed before they died or would present with disseminated disease [28,29]. Patients who are geographically isolated are also less likely to be referred to specialist centres [29], or receive adjuvant treatment [30,31] and report higher levels of unmet supportive care needs [16].

Although numerous approaches and service delivery models have been trialled in rural and regional areas of Australia, there has been inadequate evaluation of these initiatives, which would facilitate the establishment of evidence-based service models [32]. However, a recent Australian budgetary commitment to build a network of up to ten best practice regional cancer centres and accommodation sites has the potential to address the gap in access to cancer services for people living in rural and regional areas [33].

Regardless of commitments to build regional cancer centres, there is still the issue of a pervasive Australian medical workforce shortage in almost every category [34]. In some areas of New South Wales, waiting times to see an oncologist have been as long as 5 months after their initial GP appointment [35], significantly longer than the NSW health waiting time limit of 2 weeks [36] for initial specialist consultation.

The most common health care problem identified in a NSW population-based survey however, was difficulty obtaining a GP appointment [37]. The shortage of general practitioners is especially acute in rural areas. In 'large rural centres' the supply rate was 13% below that of 'capital cities' whereas 'small rural centres' and 'other rural areas' had supply rates of 23% and 35% respectively less than 'capital cities' [38]. The physician supply shortage in rural and regional areas is not isolated to Australia, but is an issue in many countries [39]. High workload and a lack of time available was another workforce issue reported by most clinicians which hindered their ability to devote appropriate amounts of time to individual patients. There is therefore, an immediate need for innovative recruitment and retention strategies to increase health professional supply. Telemedicine, as a means of providing clinical advice and consultation as well as education and training for staff, is one potential method which could have a positive impact. It allows those in rural and regional areas to access specialist advice without the need to travel [39]. Although telemedicine facilities exist in rural health services, they are predominantly used for administrative purposes [40], and therefore should be utilised to the full extent of their capabilities.

### Limitations

We acknowledge a number of limitations to this study. The response rate of care coordinators is low (33%); however this is not unusual for health professions [41]. Despite this low response rate, care coordinators composed a large percentage of the sample and therefore may over-represent their views. Other groups (general practitioners and carers) may have been under-represented. Despite this, common themes emerged from all groups. There may also be an underrepresentation of carers in our sample.

## Conclusions

This study has identified recognising health professional roles and responsibilities, implementing comprehensive MDT meeting, transitioning of care: falling through the cracks, inadequate communication between specialist and primary care, inequitable access to health services and managing scarce resources as the key barriers to coordination of cancer care. Development and evaluation of interventions based on these findings is now required to improve the quality of cancer care.

## Competing interests

The authors declare that they have no competing interests.

## Authors' contributions

JW carried out the interviews and focus groups, transcribed the individual interviews, analysed the data and drafted the manuscript. JDH assisted with focus groups, assisted with analysis of the data and helped draft the manuscript. JMY conceived of the study, participated in its design and coordination and helped to draft the manuscript. PNB assisted with the analysis of the study and helped draft the manuscript. MJS conceived of the study and helped draft the manuscript. LM assisted with the analysis of the data. All authors read and approved the final manuscript.

## Pre-publication history

The pre-publication history for this paper can be accessed here:

http://www.biomedcentral.com/1472-6963/10/132/prepub
